# Patient-specific stomach biomechanics before and after laparoscopic sleeve gastrectomy

**DOI:** 10.1007/s00464-022-09233-7

**Published:** 2022-04-22

**Authors:** Ilaria Toniolo, Alice Berardo, Mirto Foletto, Claudio Fiorillo, Giuseppe Quero, Silvana Perretta, Emanuele Luigi Carniel

**Affiliations:** 1 Department of Industrial Engineering, University of Padova, Padova, Italy; 2 Centre for Mechanics of Biological Materials, University of Padova, Padova, Italy; 3 Department of Civil, Environmental and Architectural Engineering, University of Padova, Padova, Italy; 4 Department of Biomedical Sciences, University of Padova, Padova, Italy; 5 Bariatric Surgery Unit, Azienda Ospedaliera, University of Padova, Padova, Italy; 6 Digestive Surgery Unit, Fondazione Policlinico Universitario Agostino Gemelli IRCCS, Rome, Italy; 7 Catholic University of Sacred Heart of Rome, Rome, Italy; 8 IHU Strasbourg, Strasbourg, France; 9 IRCAD France, Strasbourg, France; 10 Department of Digestive and Endocrine Surgery, NHC, Strasbourg, France

**Keywords:** Patient-specific model, Bariatric surgery, Laparoscopic sleeve gastrectomy, Biomechanics, Computational modeling

## Abstract

**Background:**

Obesity has become a global epidemic. Bariatric surgery is considered the most effective therapeutic weapon in terms of weight loss and improvement of quality of life and comorbidities. Laparoscopic sleeve gastrectomy (LSG) is one of the most performed procedures worldwide, although patients carry a nonnegligible risk of developing post-operative GERD and BE.

**Objectives:**

The aim of this work is the development of computational patient-specific models to analyze the changes induced by bariatric surgery, i.e., the volumetric gastric reduction, the mechanical response of the stomach during an inflation process, and the related elongation strain (ES) distribution at different intragastric pressures.

**Methods:**

Patient-specific pre- and post-surgical models were extracted from Magnetic Resonance Imaging (MRI) scans of patients with morbid obesity submitted to LSG. Twenty-three patients were analyzed, resulting in forty-six 3D-geometries and related computational analyses.

**Results:**

A significant difference between the mechanical behavior of pre- and post-surgical stomach subjected to the same internal gastric pressure was observed, that can be correlated to a change in the global stomach stiffness and a minor gastric wall tension, resulting in unusual activations of mechanoreceptors following food intake and satiety variation after LSG.

**Conclusions:**

Computational patient-specific models may contribute to improve the current knowledge about anatomical and physiological changes induced by LSG, aiming at reducing post-operative complications and improving quality of life in the long run.

Obesity has been defined by the World Health Organization (WHO) as a global epidemic, which affects people starting by early age and poses additional risks for related comorbidities, such as hypertension, diabetes mellitus, Nonalcoholic Fatty Liver Disease and Nonalcoholic Steatohepatitis, and lastly COVID-19 among the others [1–8].

The increasing prevalence of obesity prompted a spiraling growth of bariatric procedures, doubled from 2008 to 2018 (in which 700,000 bariatric operations were performed worldwide [9]), due to the efficacy in terms of weight loss results at least in the short-term period [10–15], even with significant costs [15–17]. Different surgical approaches have been developed, refined, or abandoned throughout the past twenty years [12, 18], and, among them, the Laparoscopic Sleeve Gastrectomy (LSG) has seen a continuous growth, being considered relatively simple (compared to other metabolic ones) but effective [15, 18–21]. However, additional concerns may arise for patients subjected to LSG, such as the gastroesophageal reflux disease (GERD) [22, 23], weight regain [10], metabolic complications [11, 13], and thus compromising the reputation in the long run.

These issues make bariatric surgery a key emerging field in biomechanics [18], in which the development of mathematical and computational models of the stomach should pave the way for detailed in silico studies of the gastric mechanics. When referring to the investigation of post-surgical behaviors, computational models may support bariatric procedures [24–28], by providing additional information, such as mechanical measurements (e.g., elongation strain distribution), or clinical ones (e.g., His angle). In particular, the design and application of patient-specific computational models could open new horizons for a better tailoring of these operations, while reducing/avoiding negative effects.

Patient-specific computational models have been recently used in different clinical fields [28–30] and are now currently performed, as they allow an ad hoc prediction of outcomes for individual patients, even though patient-specific modeling is rarely used for clinical decision making. Imaging technologies, such as Magnetic Resonance Imaging (MRI) or Computerized Tomography (CT) scans, can contribute to create and develop patient-specific geometries for several applications [31, 32], although the development of reliable and predictive models is not straightforward, since it depends on many variables such as material parameters and interactions/contacts that must be customized on the patient too.

Referring to the digestive tract, few work articles focused on the 3D geometry of the esophagus and stomach starting from medical imaging to simulate the surgical procedure in order to improve the outcomes [33, 34]. Moreover, the stomach was previously studied with finite elements approaches, but some limitations, as simplified average geometries and/or material properties derived from mechanical tests on animal samples [25, 35–37] were adopted. The lack of models as computational tools for the physio-mechanical functional investigation of digestive organs is still to overcome and gives some room for further studies and improvements.

Therefore, our aim was to develop patient-specific computational models from MRI scans of patients with morbid obesity who underwent LSG, and thus quantify the effects in terms of volumetric capacity and mechanical elongation of the gastric walls. In order to achieve clinically valuable models, the changes induced by bariatric surgery to gastric mechanical response (stiffness, volumetric capacity, and distension of gastric wall) were represented and simulated. The rational quantification of the solicitation of gastric mechanoreceptors during food intake could be the key factor in losing weight and maintaining a satisfactory quality of life because it is related to satiety cascade [38].

## Materials and methods

This study represents the elaboration in terms of computational models of the patients’ cohort reported by Quero et al. [22], adding a mechanical description of the changes in stomach geometry and solicitation of gastric wall induced by LSG. Twenty-three adult patients were treated with LSG for morbid obesity (details reported in Table 1) and all the patients underwent to MRI scans before and after bariatric surgery.

**Table 1:** Characteristics of the patients (HTN: Hypertension)

#	Age [y]	Gender	Pre-surgical Weight [kg]	Post-surgical Weight [kg]	Pre-surgical BMI [kg/m^2^]	Post-surgical BMI [kg/m^2^]	Comorbidities
01	43	M	143	98	45.13	30.93	HTN
02	54	F	93	73	41.33	32.44	Apnea-diabetes
03	24	F	100	70	33.41	23.38	–
04	40	M	132	103	37.34	29.14	HTN-reflux
05	29	F	155	108	51.19	35.67	–
06	43	M	156	135	43.21	37.39	HTN- dyslipidaemia—gonarthrosis
07	26	F	107	86	37.91	30.47	HTN- gonarthrosis
08	38	M	125	85	38.58	26.23	HTN- apnea
09	42	M	119	88	39.30	29.06	Disc herniation
10	22	F	134	95	45.29	32.11	–
11	27	F	130	95	52.07	38.05	–
12	22	F	106	82	38.93	30.11	–
13	29	F	111	82	45.03	33.26	Gonarthrosis—deep vein thrombosis
14	44	M	135	103	45.6	34.81	Gonarthrosis—dyslipidemia
15	40	F	113	92	40.5	32.98	Crohn’s disease
16	27	F	109	79	41.02	29.73	Gonarthrosis—asthma allergies
17	24	F	113	88	42.53	33.12	Polycystic ovary
18	33	F	148	109	59.28	43.66	–
19	43	F	95	75	35.32	27.88	HTN- apnea-diabetes
20	37	F	125	99	43.25	34.25	–
21	37	F	128	95	40.85	30.32	Hypothyroidism
22	31	F	136	107	42.92	33.77	–
23	58	F	100	80	39.06	31.25	HTN- apnea-diabetes

### Surgical procedure

Surgery was carried out from December 2013 to November 2015 at the Digestive Surgery Unit of the Nouvel Hôpital Civil (NHC) of Strasbourg with the support of the Image-Guided Surgery Institute (Institut de Chirurgie Guidée par l’Image/IHU) of Strasbourg and was part of a prospective, single-center study, approved by the National Ethics Committee and registered (clinicaltrials.gov Identifier: NCT01980420). The cohort comprehended 23 patients suffering from morbid obesity. All operations were performed by one of three senior bariatric surgeons, respecting the same surgical sequences and steps. The dissection of the gastroepiploic arcade along the greater curvature started from 5 to 6 cm proximally to the pylorus and ended with the division of the short gastric vessels. A calibration tube of 32 French was used and located along the lesser curvature of the stomach.

### Processing of human MRI scans and virtual solid models

**Fig. 1:**
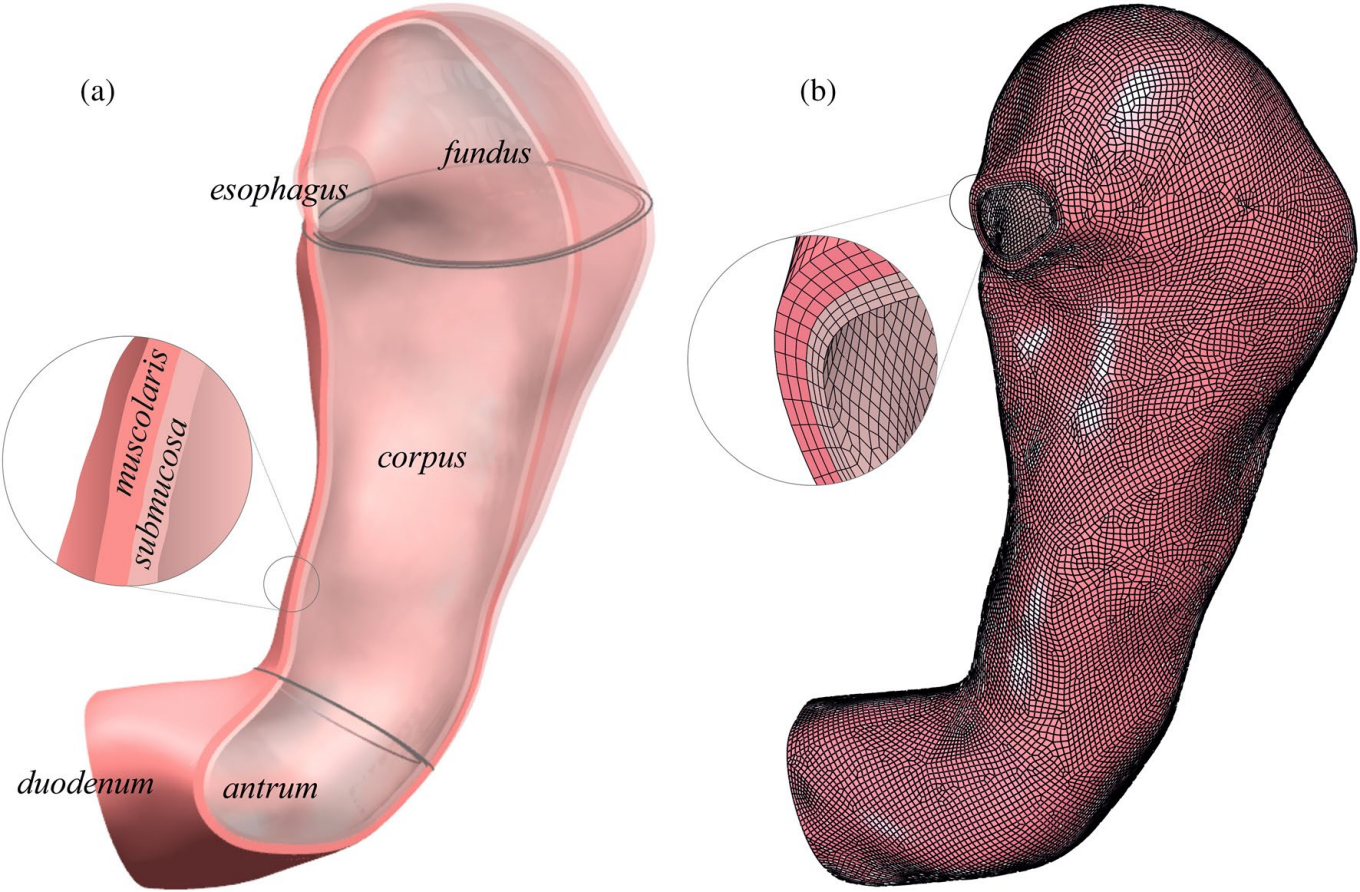
The development of a patient-specific stomach model for finite element simulations. **a** From MRI the inner volume of the stomach is extracted; then the submucosa and the muscularis layer are created by means of surface offsets, with different thicknesses depending on the specific gastric region and layer. **b** Fine elements mesh of both the layers: six hexahedral elements modeled both the submucosa-mucosa layer and the muscularis stratum along the thickness direction

For each patient two models were reconstructed, i.e., the physiological (pre-surgical) stomach and the corresponding sleeved stomach at six months after surgery. The segmentation of the MRI scans led to the generation of 46 virtual solid stomach models (#23 pre-surgical stomachs + the corresponding #23 sleeved stomachs) by means of Synopsys Simpleware ScanIP. The specifics of the scanner machine and image resolution are reported in [22]. The volumetric identification was done from the MRI sequences of an empty stomach in the transverse plane, considering the optimal view for the recognition of gastroesophageal junction. The segmentation considered the whole stomach to the pyloric ring. The stomach volumes obtained were checked in the coronal plane and then exported. A double-layered geometry was generated from subsequent offsets of the external stomach surface, to obtain the submucosa-mucosa and muscularis layer (Fig. 1a) by means of Solidworks (Dassault Systemes, 2018). Different constant thicknesses were assigned to the submucosa-mucosa and muscularis layer, according to the considered gastric region, such as 0.9 and 1.2 mm in the fundus, 1.2 and 1.5 mm in the corpus, and 0.9 and 1.8 mm in the antrum, respectively [37, 39] (Fig. 1a).

### Finite element model and simulation

For the mechanical description of patient-specific stomach behavior, every double-layered stomach geometry was imported in the numerical solver Abaqus Explicit 2018 (Dassault Systemes Simulia Corp., Providence, RI) and then discretized in a fine mesh of linear hexahedral elements of 1-mm edge size (as reported in Fig. 1b), resulting in about 175,000 nodes for pre-surgical models and 75,000 nodes for sleeved models. To obtain a better description of the gastric wall elongation during these analyses, the thickness of the two layers was discretized with at least 5 internal nodes (3 elements for the submucosa-mucosa layer and 3 elements for the muscularis layer), as shown in the details of Fig. 1b. The mechanical behavior of the stomach tissues was defined by means of a fiber-reinforced hyperelastic constitutive formulation, which included the tissue anisotropy and nonlinear elasticity. The identification of human gastric parameters was performed by means of a coupled experimental and computational approach [39], where human resected stomachs were tested at tissue, sub-structural levels, and structural levels by tensile, membrane indentation, and inflation tests, respectively. From the tensile tests a set of preliminary constitutive parameters were extracted, then optimized and validated by comparing the other experimental results with computational outputs. Both the complete formulation and the procedure for parameters identification are fully reported in previous works [25, 35, 39]. The constitutive parameters adopted in this work for the two gastric layers belonging to the three stomach regions are shown in Table 2. Since during the LSG procedure the fundus is completely removed [40], when modeling sleeved stomachs, this region was omitted, and post-surgical models were described only by the corpus and the antrum, with related constitutive parameters.

**Table 2:** Constitutive parameters adopted in the mechanical formulation of the stomach tissue

Region	Layer	*C*_1 _[kPa]	*α*_1_ [−]	*C*_4_ [kPa]	*α*_4 _[−]	*C*_6_ [kPa]	*α*_6_ [−]
Fundus*	Submucosa-mucosa	0.15	1.05	3.30	0.96	3.70	1.11
	Muscularis	0.15	1.05	5.20	0.68	7.10	0.70
Corpus	Submucosa-mucosa	0.15	1.05	3.00	1.80	3.00	1.68
	Muscularis	0.15	1.05	10.09	0.24	9.70	0.23
Antrum	Submucosa-mucosa	0.15	1.05	4.50	0.11	3.00	0.54
	Muscularis	0.15	1.05	3.01	0.39	4.50	0.54

*Since the fundus part is completely removed after the LSG, its parameters were not included in the mechanical behavior of the only sleeved stomach models

The assessment of the changes induced by bariatric surgery focused on gastric volumetric reduction, gastric mechanical response of the stomach after a simulated inflation (pressure–volume curve), and the distribution of elongation strain at different levels of intragastric pressure. Hence, to simulate an inflation process and thus characterize the total deformation of the gastric walls mimicking the process of food ingestion, a fluid cavity interaction was defined in the internal region of the stomach, while gastroesophageal and gastroduodenal junctions were fixed by imposing null displacement to the upper and lower extremities of the stomach cavity. Each computational analysis was performed by progressively increasing the intracavity pressure up to 40 mmHg during a step time of 1 s. The simulations lasted between 12 and 24 h, running contemporary on 45 threads of a High-Performance Computing Server Fujitsu Primergy RX4770 equipped with two Intel Xeon E7 8890 v4 processors, 256 GB RAM and SSD HD.

The validation of the obtained results was performed by comparing the experimental volumes and pressures of the pre- and post-surgical stomachs obtained in [22] with the computational predictions in the same situation, as reported in Fig. 3.

### Statistical analysis

The 2-samples t-test was carried out considering the volumetric capacity (at baseline and at 30 mmHg), the elongation strain (at 20, 30, and 40 mmHg), and the variation of elongation strain both in pre- and post-surgical configurations among the following categories: gender (6 males and 17 females), obesity severity (“no obese”, I, II, and III obesity classes) in pre-surgical (0, 1, 7, and 15 individuals, respectively), and post-surgical (6, 13, 3, and 1 individuals) patients, age (up to 40 years and over 40, resulting in sixteen < 40-year-old and seven > 40-year-old individuals), and presence of comorbidities (“yes” or “no,” 15 and 8, respectively). The limit to classify the age was imposed equal to (maximum age − minimum age)/2 + minimum age. For these tests, the standard significance level α was considered equal to 5%.

Paired t-tests were performed when comparing the pre- and post-surgical quantities which are referred to the same patient, therefore they cannot be considered “independent” samples, i.e., volumetric capacity and principal logarithmic elongation strain (namely ES) with respect to increasing intragastric pressure (baseline and 30 mmHg for the volume and 20, 30, and 40 mmHg for ES).

## Results

The analyzed patients’ cohort is characterized by mean age 35 (± 9.9), pre-surgical mean weight 122.30 (± 18.55) kg associated to a pre-surgical BMI of 42.57 (± 1.2) kg/m^2^, while the post-surgical weight was 92.48 (± 14.67) kg associated to a post-surgical BMI of 32.18 (± 4.22) kg/m^2^, respectively.

The computational results are presented in terms of volumetric capacity (pressure–volume response) and distension of gastric wall (elongation stain) for the 23 pre-surgical stomachs and the corresponding 23 sleeved stomachs.

The elongation strain (ES) represents a variation of the total elongation (intended as the difference between the initial and final length) with respect to the initial configuration, and usually is expressed as percentage of this latter.

**Fig. 2:**
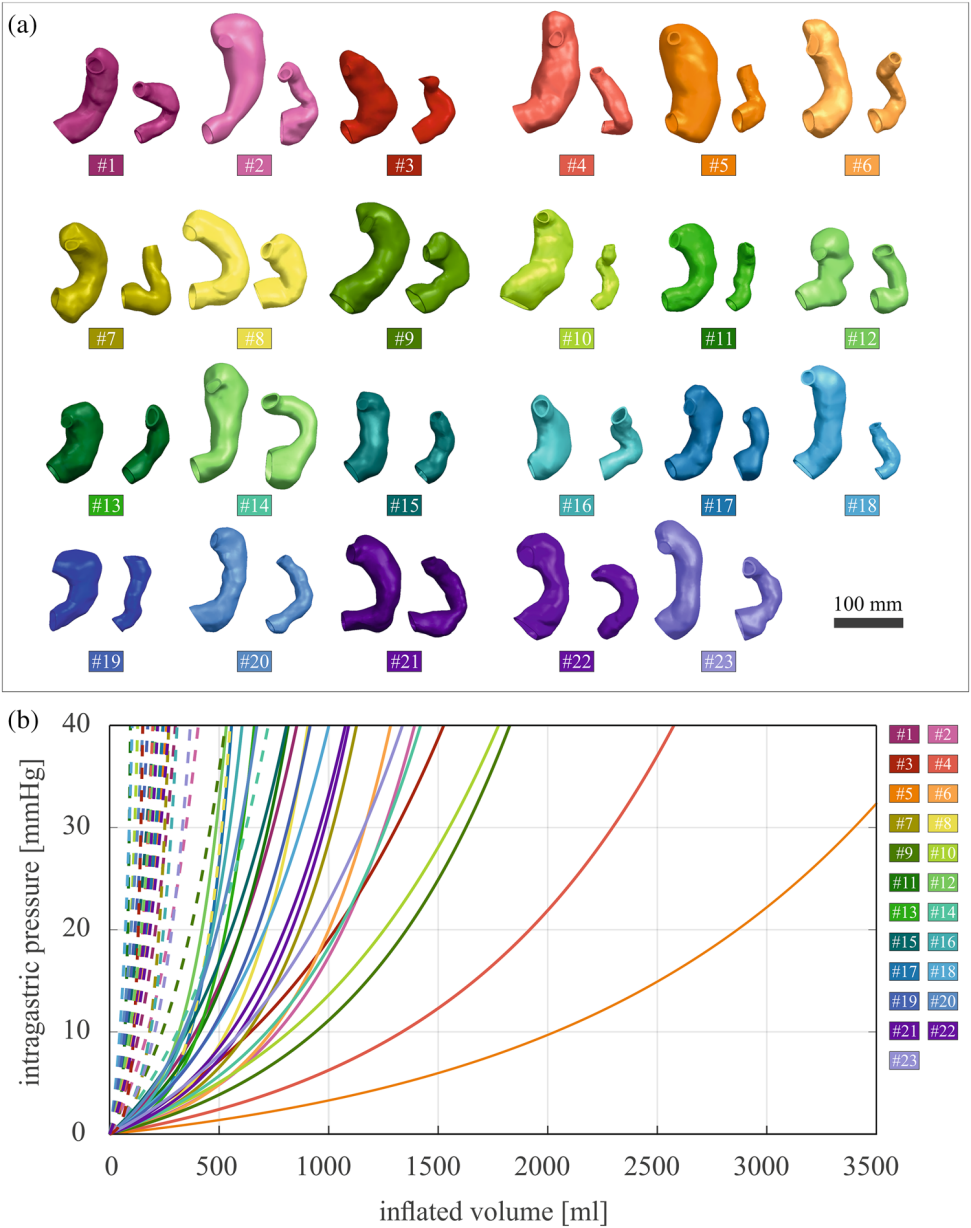
**a** Patient-specific models obtained from the MRI of the 23 considered patients, before and after LSG. The scale bar is 100 mm. **b** Pressure–volume relationships obtained after an inflation simulation; solid lines represent the pre-surgical stomachs, dashed lines stated for the post-surgical ones

**Fig. 3:**
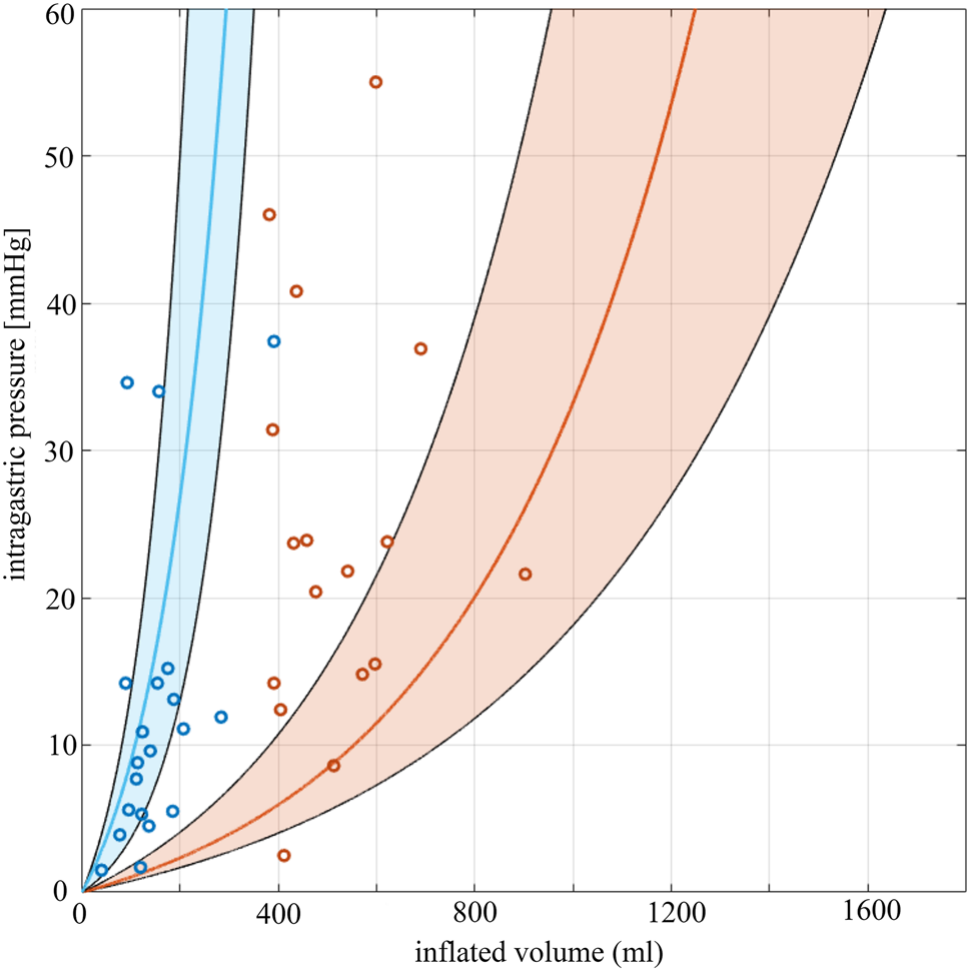
The statistical bands (C.I. 75%) and the corresponding median curves of computational pre-surgical (orange curve) and sleeved (blue curve) stomachs are reported. Results are compared with experimental results, where orange circles and blue circles represent pre- and post-surgical measurements, respectively

Figure 2a shows the extracted patient-specific stomach models (from patient #1 to #23, pre- and post-surgery), and in Fig. 2b the pressure–volume response of each model from 0 to 40 mmHg has been extracted from the computational simulations. The solid lines describe the pre-surgical stomachs during the inflation process, while the dashed lines account for the sleeved stomachs. These latter display a smaller variability, to be expected after LSG, due to the final reduced stomach volume, approximatively the same in all the patients at least in the short term. In particular, as reported in [22], a calibration tube of 32 Fr bougie size was used. In addition, the statistical bands (C.I. 75%) and the corresponding median curves of pre-surgical stomachs and sleeved stomachs are shown in Fig. 3. The computational outputs in terms of volumetric capacity were firstly compared with experimental volumes, to validate the feasibility of the 3D finite element model generation procedure, highlighting no significant differences between the two groups (*p* = 0.14). Then, experimental volumes and related intragastric pressures obtained through manometry in [22] were plotted with respect to the computational predictions. In Fig. 3, orange circles and blue circles represent pre- and post-surgical measurements, respectively.

Table 3 reports the collection of the computational volumetric capacity of each patient at baseline, i.e., at zero intragastric pressure, which identifies the dimension of empty stomachs, and the reached volume at an inner intragastric pressure of 30 mmHg, which represents a realistic physiological pressure occurring during a meal.

**Table 3:** Baseline volumes and inflated volumes (at 30 mmHg) extracted from the finite element models at baseline and at 30 mmHg of intragastric pressure

#	Baseline pre-surgical volume [ml]	Baseline post-surgical volume [ml]	Pre-surgical volume at 30 mmHg [ml]	Post-surgical volume at 30 mmHg [ml]
01	136.69	43.83	710.91	257.77
02	252.33	50.19	1265.96	344.92
03	171.81	31.37	1290.94	144.08
04	327.75	38.30	2291.39	176.90
05	519.68	35.28	3400.93	216.57
06	187.84	38.09	1134.50	229.26
07	192.47	56.81	1024.04	258.83
08	152.80	91.90	834.42	524.52
09	237.61	67.61	1641.88	455.04
10	194.64	18.19	1527.75	114.12
11	114.98	27.62	716.06	89.54
12	99.86	33.89	488.75	182.08
13	137.96	44.71	621.00	207.26
14	232.55	98.36	1250.72	616.15
15	112.51	32.46	691.98	166.57
16	112.11	42.54	551.65	230.61
17	130.87	42.15	500.24	209.51
18	148.42	19.50	866.34	88.44
19	113.04	23.58	804.62	146.29
20	84.708	24.87	588.39	126.33
21	146.10	49.21	955.86	286.54
22	143.68	37.96	983.92	191.31
23	168.99	50.27	1147.53	334.49
Mean (± St.dev)	179.10 (± 93.6)	43.43 (± 20.19)	1100 (± 658)	243.4 (± 135.1)

**Fig. 4:**
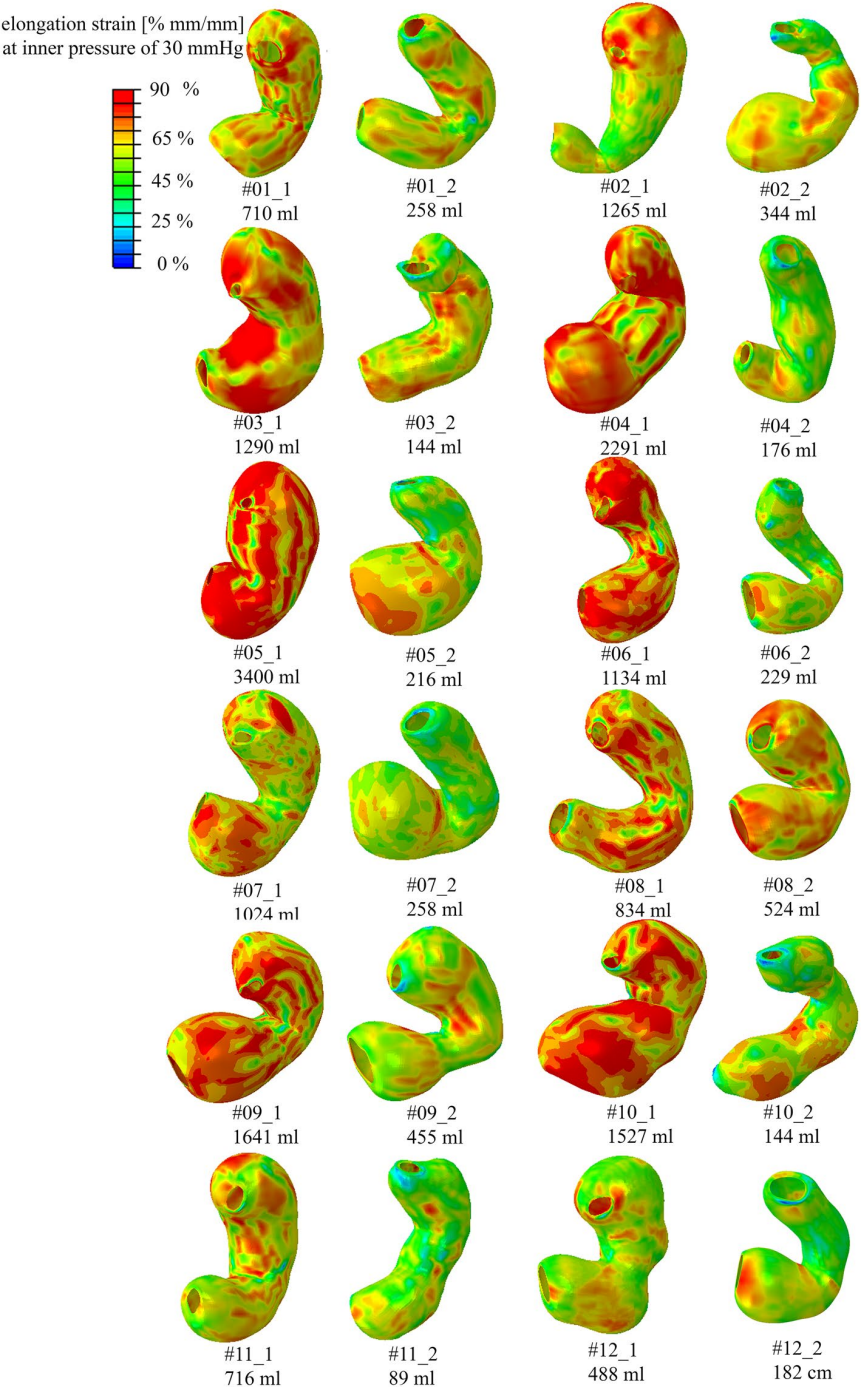
Patients from #01 to #12: colormaps of the distribution of elongation strain for pre-surgical stomachs and sleeved stomachs with applied inflation pressure of 30 mmHg (about 4 kPa). The symbols “_1” and “_2” indicate the pre-surgical and post-surgical stomach configurations, respectively. Please note that the models are not in scale

**Fig. 5:**
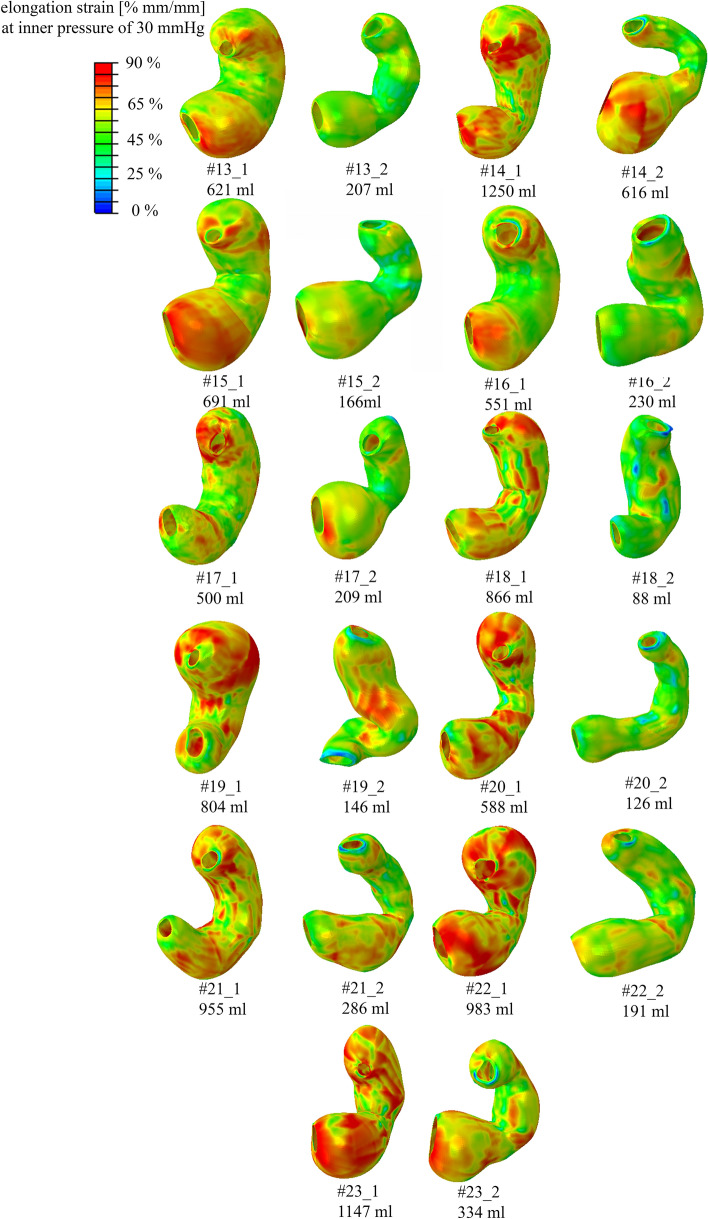
Patients from #13 to #23: colormaps of the distribution of elongation strain for pre-surgical stomachs and sleeved stomachs with applied inflation pressure of 30 mmHg (about 4 kPa). The symbols “_1” and “_2” indicate the pre-surgical and post-surgical stomach configurations, respectively. Please note that the models are not in scale

For each model, the distribution of ES was exported. The colormaps of these distributions, referring to the gastric wall during the inflation process, are reported in Fig. 4 and Fig. 5 (not in geometrical scale) for pre-surgical and sleeved stomachs, when the models reached an inner intragastric pressure of 30 mmHg, highlighting that the LSG procedure induced a decrease in the elongation strain of the whole model.

**Fig. 6:**
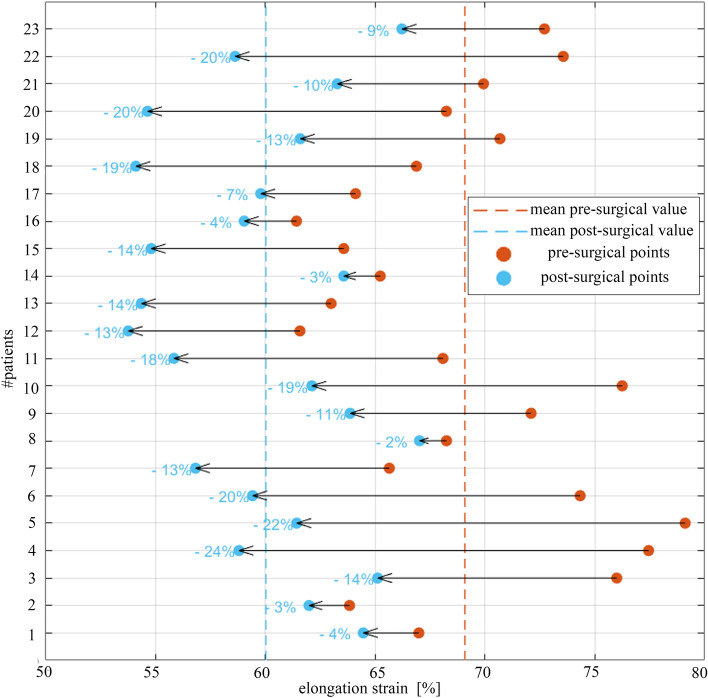
Reduction of the average elongation strain with respect to pre-surgical values for each patient-specific model. Red dashed line states for the mean strain value of pre-surgical models at 30 mmHg, blue dashed line represents the mean strain value of the post-surgical models, at the same applied pressure

To rationally quantify the difference in terms of ES developed on gastric wall, the mean values of the whole models are plotted in Fig. 6. For each patient, ES variation was calculated as $\left[ {\left( {{\text{post}} - {\text{surgical elongation }}{-}{\text{ pre}} - {\text{surgical elongation}}} \right)/{\text{ pre}} - {\text{surgical elongation}}} \right] \, *{1}00$ and reported in light blue. On average for stomachs at 30 mmHg pressure, a variation of ES of 13% was observed after LSG.

**Fig. 7:**
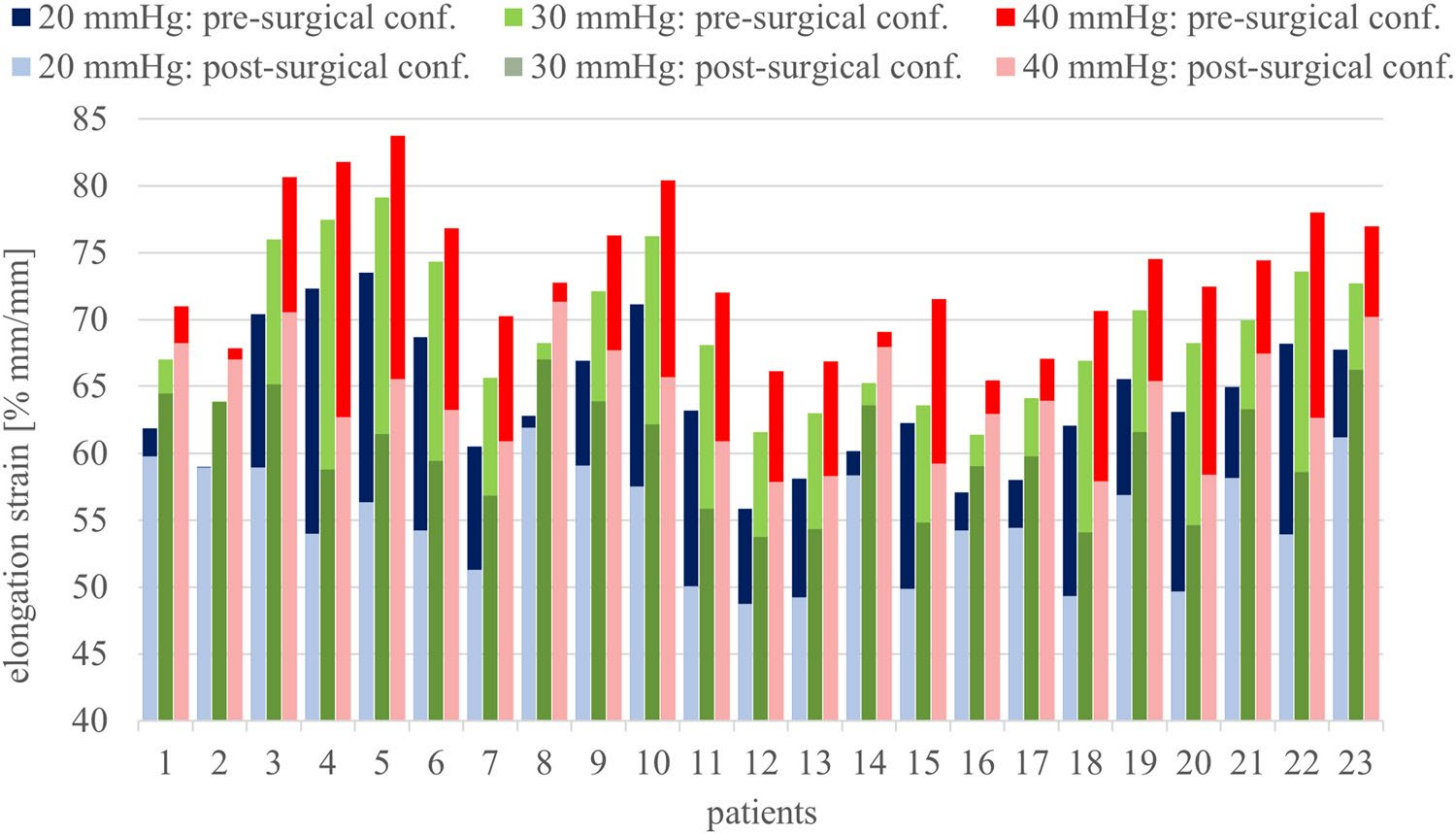
Average elongation strain (ES) for each pre- and post-surgical models obtained at different intragastric pressure levels (blue: 20 mmHg, green: 30 mmHg, red: 40 mmHg). For each level, overlapping full and light color bars account for pre- and post-surgical LE, respectively

For a better comparison of pre- and post-surgical results, the average ES values were also extracted at other pressure levels (with reference to those obtained in [22]), i.e., 20 and 40 mmHg (Fig. 7). For each level, full and light color columns, referred to pre- and post-surgical ES, were plotted overlapped. There is a significant difference of ES behavior, clearly reported by the reduction percentages, with peaks up to 24%. This behavior is clearly visible in Fig. 7, highlighting bigger ES in all the pre-surgical conformations.

Age category impacted on post-surgical ES at 40 mmHg (*p* = 0.019), resulting in higher statistical values of ES in patients > 40 years old (*p* = 0.012). However, patients < 40 years recorded statistical higher values of variation of ES at 20 mmHg (*p* = 0.035).

The presence or not of comorbidities influenced statistically (*p* = 0.001) the post-surgical volumetric capacity, leading to a significantly minor volumetric capacity after LSG, at baseline and 30 mmHg of intragastric pressure, in patients without comorbidities (*p* = 0.003). Moreover, comorbidities category had an impact on the variation of ES (at 20, 30, and 40 mmHg of intragastric pressure), revealing that patients without comorbidities would be characterized by a greater ES variation (*p* = 0.003, *p* = 0.002, *p* = 0.003, respectively).

Considering the gender, male patients showed greater values in post-surgical capacity at baseline and at 30 mmHg (*p* = 0.002 at baseline vs *p* = 0.001 at 30 mmHg).

Significant differences were recorded when pre- and post-surgical outcomes were considered. In fact, volumes resulted statistically greater before LSG than after (*p* < 0.0001), and this observation can be extended to ES, as well, at different pressure levels (*p* < 0.0001). The statistical results are summarized in Tables 4 and 5.

**Table 4:** Results of the statistical 2-sample t-tests. Only the significant results were reported

Quantity	#	Category	Mean (± std)	*p* value (µ_1_- µ_2_ ≠ 0)	*p* value (µ_1_ < or > µ_2_)
Post-surgical ES at 40 mmHg	7	> 40 years old	67.33 (± 2.3) %	0.019	0.012
	16	< 40 years old	62.89 (± 4.3) %		
Post-surgical volumetric capacity at baseline	8	No comorbidity	28.59 (± 7.31) ml	0.001	0.003
	15	At least one comorbidity	51.3 (± 20.5) ml		
Post-surgical volumetric capacity at 30 mmHg	8	No comorbidity	144.1 (± 48.1) ml	0.001	0.003
	15	At least one comorbidity	296 (± 137) ml		
Post-surgical volumetric capacity at baseline	17	F	36.5 (± 11.6) ml	0.003	0.002
	6	M	63 (± 27.2) ml		
Post-surgical volumetric capacity at 30 mmHg	17	F	196.3 (± 77.5) ml	0.003	0.001
	6	M	377 (± 180) ml		
Variation of ES at 20, 30, and 40 mmHg	15	At least one comorbidity	10.72 (± 2.3) % 9.96 (± 6.61) % 9.55 (± 6.7) %	*p* = 0.007 *p* = 0.004 *p* = 0.007	*p* = 0.003 *p* = 0.002 *p* = 0.003
	8	No comorbidity	19.38 (± 3.36) % 18.15 (± 3.18) % 17.19 (± 3.41) %

**Table 5:** Results of the statistical paired tests. Only the significant results were reported

Quantity	Configuration	Mean (± std)	*p* value (µ_difference_ = 0)	*p* value (µ_difference_ > or < 0)
ES at 20 mmHg	Pre	64.06 (± 5.08) %	0	*p* < 0.0001
	Post	55.06 (± 4.22) %		
ES at 30 mmHg	Pre	69.1 (± 5.29) %	0	*p* < 0.0001
	Post	60.04 (4.15) %		
ES at 40 mmHg	Pre	73.33 (± 5.28) %	0	*p* < 0.0001
	Post	64.17 (± 4.23) %		
Volumetric capacity at baseline	Pre	179.1 (± 93.6) ml	0	*p* < 0.0001
	Post	43.4 (± 20.2) ml		
Volumetric capacity at 30 mmHg	Pre	1100 (± 658) ml	0	*p* < 0.0001
	Post	243.4 (± 135.1) ml		

## Discussion

Among the bariatric operations performed worldwide, LSG provides a strong reduction in the baseline volume capacity of the stomach, up to 80%. The almost total removal of fundus and the strongly changed post-surgical anatomy imply a lesser ingestible amount of food and a modified mechanical stimulation of the gastric wall, leading to different solicitation of the gastric receptors which could affect satiety [41–44]. The intervention usually results in a significant weight loss and comorbidities improvement, ameliorated patients’ quality of life [18, 20], although other issues (e.g., GERD, unsatisfactory approach to food) [22, 23] may arise, impacting on long-term results.

Furthermore, the expected mechanical behavior of stomach, in terms of ES and related stress field due to food ingestion, is quite difficult both in vivo and ex vivo. Therefore, in silico models can be a useful tool for computing gastric wall stimuli and forecasting post-surgical gastric capacity and strain distribution, especially when referring to patient-specific models.

From the extracted geometries reported in Fig. 2a and the simulated volume–pressure curves displayed in Fig. 2b, it emerges that patient-specific stomach models present a wide inter-sample variability both in shape, size, and mechanical behavior, especially in pre-surgical stomachs. This variability displays a typical feature of biological tissues and structures, more evident in pre-surgical curves, which occupied the rightmost part of the chart (related to higher volumes) with respect to the post-surgical settings [22]. These latter models exhibit a more contained variability, because of the volumetric standardization by the calibration tube (in this study 32 Fr bougie size) used for LSG, which forced the patient-specific stomach geometry to a predefined volume, with small oscillations within six months after the surgery. Indeed, statistical analysis clearly shows significant difference between pre- and post-surgical baseline volumes, also when a physiological intragastric pressure of 30 mmHg was applied. Moreover, it was possible to validate the models, when comparing experimental evidences with computational predictions, as reported in Fig. 3. In particular, computational results of post-surgical stomachs showed a good agreement with respect to experimental measurements of intragastric pressure and related volumetric capacity, while for pre-surgical models, they appeared to overestimate the gastric volumes at the same pressure, with respect to the real ones. The main reasons could be related to the adoption of simplified constant pressure, normal to each point of the internal surface (instead of different intragastric pressure levels), to model the ingestion process, and also the lack of the conformational effect of adjacent organs combined with positive intra-abdominal pressure. The first approximation may force the fundus to a hyper-physiological extension, while the second simplification affects the final volume, which resulted greater with respect to the measured one at the same intragastric pressure.

ES assessments are not so straightforward when carried out in vivo tests but ES is strictly correlated to the mechanical behavior of different stomach regions. As for the volume, the outputs of ES distribution showed a high variability between pre- and post-surgical configurations, but also within pre- and post-surgical models (as reported in Fig. 4 and Fig. 5, with no geometrical scale models). These discrepancies can be explained by two main factors: a variable patient-specific geometrical shape and the presence of different gastric areas. It is remarkable that the sole volume reduction between pre- and post-surgical stomachs does not account for the global ES reduction. The latter is influenced by the mechanical properties of the involved gastric anatomical regions, i.e., corpus and antrum. In fact, fundus has the softest mechanical parameters and is removed after LSG. This resulted in a stiffer structure, which thus displays, for a given internal pressure, a lower ES compared with a pre-surgical stomach. Moreover, every model has a different subdivision of the stomach regions due to the geometry variability, which influences the total number of elements describing the fundus, corpus, and antrum and the ratios among them, as well.

In addition, the configuration of the sleeved stomach may also play a key role in reducing the ES. According to Laplace law (the larger the vessel radius, the larger the wall tension required to withstand a given internal fluid pressure), with the same internal fluid pressure the modification of the stomach into a tubular geometry with a smaller diameter elicits a minor wall tension required for the equilibrium and then a minor ES of the gastric walls, with respect to the pre-surgical situation.

ES analyses at increasing internal pressure (Fig. 6 and Fig. 7) confirmed this trend, with bigger ES in all the pre-surgical geometries.

In support of the computational analyses, the statistical tests confirmed significant differences introduced by the LSG procedure, with post-surgical data (like volumetric capacity and elongation strain) statistically changed after surgery.

From a biomechanical point of view, several studies have linked the presence of other pathologies, such as diabetes or hypertension, with a change in the mechanical properties of tissues [45–50], supporting our hypothesis that comorbidities could affect the stomach tissue mechanical behavior, also in the post-surgical configuration. On the contrary, from a clinical perspective, this observation has not been investigated yet, thus suggesting a new starting point for further medical investigations. In our study, comorbidities appeared to influence the elongation behavior at different internal pressure levels (20, 30, and 40 mmHg) since the statistical analyses pointed out that patients with comorbidities were characterized by a smaller ES variation that in some cases also correlated to patients’ age.

No statistical differences were found as far as volumetric capacity differences, confirming that the ES is not necessarily linked to volumetric capacity, but to other factors.

Similar but preliminary results were reported by Toniolo et al. [37] that represented the LSG stomach with cylindrical models assuming no sample variability, which on the opposite was evident in this work. However, some assumptions were also adopted in current analyses, such as the absence of gastric emptying, gastric motility, and the fluid–structure interaction between gastric wall and the bolus, simplified as water content.

Some limitations refer to the adoption of a constant intragastric pressure and the lack of the surrounding organs which could lead to an overestimation of the final volume of the inflated stomach, especially for the pre-surgical ones (where the fundus is considered).

Moreover, when dealing with LSG surgery, the stapler line could slightly influence the pressurization due to the higher stiffness of metallic clips, and thus influence the overall stiffness of the biological tissues. This may cause a different strain distribution, especially close to the clips, with a consequent higher stress concentration. This aspect was not considered in post-surgical models, leading to possible overestimates of the ES. For these reasons, future developments will focus also on this point, to evaluate possible high stress concentrations and consequent tissues degeneration. Despite these simplifications, we can infer that the feasibility of the applied computational model is assessed especially by comparison with the post-surgical pressure–volume relationships (Fig. 3), thus confirming the reliability of both the geometrical reconstruction procedure and the identification of constitutive parameters. In addition, these insights strengthen the importance of computational tools and studies for operation planning and predictions also in the bariatric surgery, a clinical and surgical area that in recent years is attracting new interest within the scientific community.

## Conclusions

Bariatric surgery is considered the best option to treat people with morbid obesity but needs to be refined, since it is mainly based on empiric approach, with sometimes complications and side effects. Computational modeling can be a powerful tool to address the main limits of bariatric surgery, without performing additional clinical trials and animal testing.

This work pointed out the importance to use a patient-specific approach for a better comprehension of the effects of LSG procedure aimed at improving bariatric surgery outcomes. Even if some assumptions were adopted, stomach mechanics showed a different behavior after the operation, mainly regarding ES field. The removal of the fundus due to the LSG resulted in a stiffer structure with important reflexes on food intake and satiety. Moreover, lower values of ES are related to the reduction in stomach diameter, which implies a minor gastric wall tension, at equal internal pressure (Laplace law).

Future developments concerning this topic will include other key variables, as the role of the gastric wall and its interaction with metallic clips, which could generate high stress concentrations and possible tissues damages.
